# The Hologenome Across Environments and the Implications of a Host-Associated Microbial Repertoire

**DOI:** 10.3389/fmicb.2017.00802

**Published:** 2017-05-11

**Authors:** Tyler J. Carrier, Adam M. Reitzel

**Affiliations:** Department of Biological Sciences, University of North Charlotte at CharlotteCharlotte, NC, USA

**Keywords:** ecology, holobiont ecology, hologenome theory, microbial repertoire, evolution

## Abstract

Our understanding of the diverse interactions between hosts and microbes has grown profoundly over the past two decades and, as a product, has revolutionized our knowledge of the life sciences. Through primarily laboratory experiments, the current framework for holobionts and their respective hologenomes aims to decipher the underpinnings and implications of symbioses between host and microbiome. However, the laboratory setting restricts the full spectrum of host-associated symbionts as compared to those found in nature; thus, limiting the potential for a holistic interpretation of the functional roles the microbiome plays in host biology. When holobionts are studied in nature, associated microbial communities vary considerably between conditions, resulting in more microbial associates as part of the “hologenome” across environments than in either environment alone. We review and synthesize empirical evidence suggesting that hosts may associate with a larger microbial network that, in part, corresponds to experiencing diverse environmental conditions. To conceptualize the interactions between host and microbiome in an ecological context, we suggest the “host-associated microbial repertoire,” which is the sum of microbial species a host may associate with over the course of its life-history under all encountered environmental circumstances. Furthermore, using examples from both terrestrial and marine ecosystems, we discuss how this concept may be used as a framework to compare the ability of the holobiont to acclimate and adapt to environmental variation, and propose three “signatures” of the concept.

## Nature of the holobiont

Partnerships between host and microbes within an environmental setting exemplify a network of biotic relationships that are common across the tree of life (Rosenberg and Zilber-Rosenberg, 2013; Bosch and Miller, 2016; Hurst, 2016). The phenomena underlying these relationships complement more than a century of biological research that has focused on the evolution and ecology of individual species. To conceptualize the functional importance between host and microbiota, Zilber-Rosenberg and Rosenberg (2008) proposed the hologenome theory of evolution, stating that animals and plants, along with their microbiome serve as a unit of selection. This paradigm shift has led to advancements in our understanding of the spectrum of organismal symbioses in the life sciences (Bordenstein and Theis, 2015; Theis et al., 2016), with particular attention to developmental (McFall-Ngai and Ruby, 2000; McFall-Ngai, 2002), evolutionary (Brucker and Bordenstein, 2012, 2013a,b), and genetic modifications (Husnik et al., 2013) to the host.

The hologenome theory emphasizes the role microbes play in animal and plant evolution as integrated units of biological organization that intertwine Darwinian and Lamarckian principles (Zilber-Rosenberg and Rosenberg, 2008; Rosenberg et al., 2009; Bordenstein and Theis, 2015). The hologenome theory provides functional explanations for the role of the microbiome in a Darwinian framework as it relates to speciation (Brucker and Bordenstein, 2012, 2013b) and potentially host fitness (e.g., Callens et al., 2016). A Lamarckian framework (Rosenberg et al., 2009), on the other hand, is complementary to this, as it details the mechanisms whereby microbes are acquired or lost during an organism's lifetime, and that the acquisition of a novel species or strain of microorganism may be integrated into the hologenome. Therefore, hologenomes (and as a direct extension, the holobiont) integrate principles from multiple disciplines (Rosenberg et al., 2009) spanning the diverse fields of evolutionary genetics (Brucker and Bordenstein, 2012, 2013b) and evolutionary ecology (Macke et al., 2016; Theis et al., 2016).

One major challenge the field currently faces is melding insights from the evolutionary, genetic, and molecular underpinnings of host-microbe partnerships with the ecological conditions in which they formed and evolved. Recent work has begun addressing these disciplines as an integrative discipline (Gilbert et al., 2015; Theis et al., 2016); however, they remain largely as separate conceptual entities. Since the hologenome concept was proposed nearly a decade ago, other multi-disciplinary fields, such as evolutionary developmental biology (Moczek et al., 2015), have recognized and, in a conceptual as well as empirical manner, addressed this challenge in two successive steps. The first of these emphasizes the value of applying hypotheses and/or mechanisms derived from laboratory studies to complementary experiments in nature (Gilbert, 2016), while the second tests evolutionary principles across different environments. Based on our current interpretation of animal-microbe partnerships, we provide an initial assessment of these two steps, whereby using published data we quantify the degree that host-associated microbiomes differ between laboratory- and field-based studies (Table 1), as well as when the hologenome faces an environmental stress (Table 2).

**Table 1:** **Representative list of studies in different animals comparing host-associated microbiota in the laboratory and field**.

**Group**	**Species**	**Study**
Cnidarian	*Fungia granulosa*	Kooperman et al., 2007
	*Hydra* spp.	Fraune and Bosch, 2007
	*Nematostella vectensis*	Mortzfeld et al., 2015
Fish	*Cyprinus carpio*	Eichmiller et al., 2016
	*Hypophthalmichthys nobilis*	Eichmiller et al., 2016
	*Hypophthalmichthys molitrix*	Eichmiller et al., 2016
	*Danio rerio*	Roeselers et al., 2011
Insect	*Aphis glycines*	Bansal et al., 2014
	*Bactrocera tryoni*	Morrow et al., 2015
	*Bactrocera neohumeralis*	Morrow et al., 2015
	*Bactrocera jarvisi*	Morrow et al., 2015
	*Bactrocera cacuminata*	Morrow et al., 2015
	*Ceratitis capitata*	Morrow et al., 2015
	*Dirioxa pornia*	Morrow et al., 2015
	*Camponotus fragilis*	He et al., 2014
	*Drosophila* (14 species)	Chandler et al., 2011
	*Helicoverpa armigera*	Xiang et al., 2006
	*Ostrinia nubilalis*	Belda et al., 2011
Lizard	*Liolaemus parvus*	Kohl et al., 2014b
	*Liolaemus ruibali*	Kohl et al., 2014b
	*Phymaturus williamsi*	Kohl et al., 2014b
Mice	*Mus musculus*	Kohl et al., 2014b
Nematode	*Caenorhabditis elegans*	Dirksen et al., 2016
Woodrats	*Neotoma albigula*	Kohl et al., 2014b
	*Neotoma stephensi*	Kohl et al., 2014b

**Table 2:** **Representative list of studies comparing host-associated microbiota between environments for different animals (please note that social environment/interactions were not addressed but may contribute to hologenomic composition, e.g., see Tung et al., 2015)**.

**Group**	**Species**	**Environmental Factor(s)**	**Study**
Amphibian	*Rana cascadae*	Habitat-type	Kueneman et al., 2014
	*Salamandra salamandra*	Habitat-type	Bletz et al., 2016
Cnidarian	*Acropora millepora*	pH, Temperature	Webster et al., 2016
	*Acropora millepora*	Temperature	Littman et al., 2011
	*Aplysina cauliformis*	Light	Freeman et al., 2015
	*Aplysina fulva*	Light	Freeman et al., 2015
	*Balanophyllia europaea*	pH	Meron et al., 2012
	*Cladocora caespitosa*	pH	Meron et al., 2012
	*Montastraea annularis*	Organic Carbon Level	Kline et al., 2006
	*Nematostella vectensis*	Salinity, Temperature	Mortzfeld et al., 2015
	*Seriatopora hystrix*	pH, Temperature	Webster et al., 2016
Coralline algae	*Hydrolithon onkodes*	Temperature	Webster et al., 2011b
	*Neogoniolithon fosliei*	Temperature	Webster et al., 2011b
Fish	*Oreochromis niloticus*	Starvation	Kohl et al., 2014a
Foraminifera	*Heterostegina depressa*	pH, Temperature	Webster et al., 2016
	*Marginopora vertebralis*	pH, Temperature	Webster et al., 2016
Frog	*Lithobates pipiens*	Temperature	Kohl and Yahn, 2016
Geckos	*Eublepharis macularius*	Starvation	Kohl et al., 2014a
Human	*Homo sapiens*	Diet-type	Turnbaugh et al., 2009
Insect	*Acyrthosiphon pisum*	Diet-type	Gauthier et al., 2015
	*Nezara viridula*	Temperature	Kikuchi et al., 2016
Mice	*Mus musculus*	Starvation	Kohl et al., 2014a
	*Mus musculus*	Diet-type	Sonnenburg et al., 2016
	*Mus musculus*	Light	Thaiss et al., 2016
Nematode	*Caenorhabditis elegans*	Organic Matter (Soil)	Berg et al., 2016
Primates	*Gorilla gorilla gorilla*	Diet-type	Gomez et al., 2015
Quail	*Coturnix coturnix*	Starvation	Kohl et al., 2014a
Sea urchin	*Echinometra* sp.	pH, Temperature	Webster et al., 2016
Sponge	*Axinella corrugata*	Season	White et al., 2012
	*Rhopaloeides odorabile*	Temperature	Webster et al., 2011a
Toads	*Anaxyrus terrestris*	Starvation	Kohl et al., 2014a
Woodrats	*Neotoma bryanti*	Diet-type	Kohl and Yahn, 2016
	*Neotoma lepida*	Diet-type	Kohl and Yahn, 2016

## Toward nature's laboratory

Traditional animal models (e.g., *Hydra*: Fraune and Bosch, 2007; *Drosophila*: Shin et al., 2011; *Mus*: Sonnenburg et al., 2016) are powerful systems for laboratory experiments, particularly when dissecting the molecular mechanisms of organism-level processes under controlled settings. Substantial research with these species has led to fundamental discoveries in the formation, regulation, and diversity of microbial symbioses (e.g., Ley et al., 2005; Turnbaugh et al., 2006, 2009). However, these species, or any other for that matter, studied in the laboratory only partially represent the full spectrum of associations they may have with microbes in the natural setting where they have evolved (Chandler et al., 2011). In addition, species reared in the laboratory for many generations may have undergone artificial selection, intended or not, wherein phenotypic and genotypic traits having been selected for in response to abiotic and biotic environmental pressures may have been modified or lost (Kiers et al., 2007). As others have acknowledged before (e.g., Chandler et al., 2011; Har et al., 2015; Dirksen et al., 2016; Table 1) and as we do here, to what extent does the microbiome of laboratory animals reflect that of wild counterparts?

**Figure 1:**
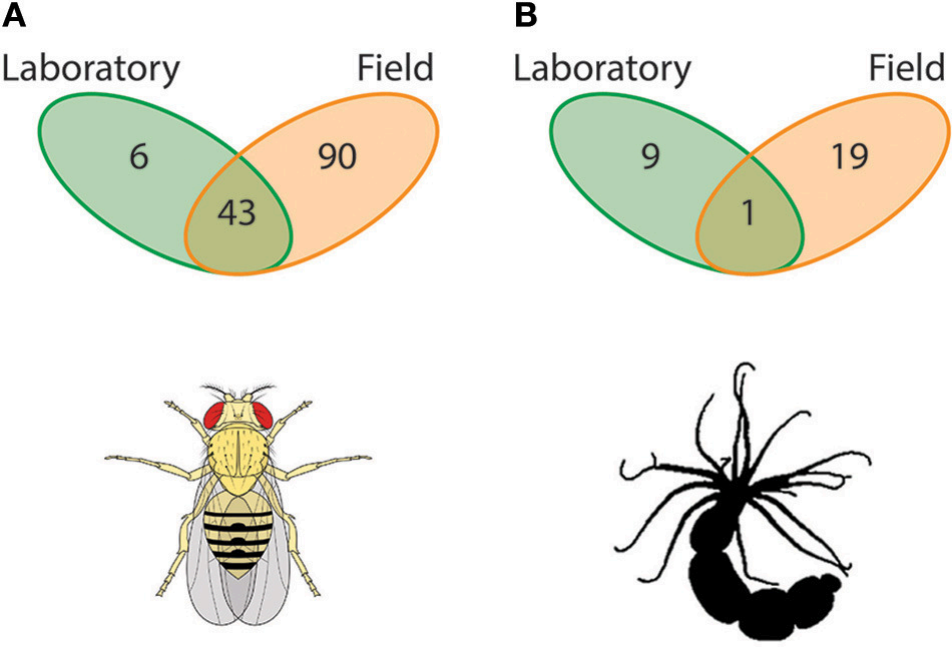
**Host-associated microbial symbionts in laboratory vs. field**. An overwhelming majority of host-associated microbiome studies have been conducted in the laboratory. This artificial and highly regulated setting inhibits the host from maintaining symbioses with the diversity of microbes that they do in the field, a phenomenon found broadly across the animal kingdom. Here, we present data from Chandler et al. (2011) and Har et al. (2015) that compared the microbiome of laboratory-entrained and wild caught Drosophilids and starlet sea anemone *Nematostella vectensis*. **(A)** For the *Drosophila* spp. system, there are a total of 139 associated OTUs in these settings with 90 specific to wild individuals, six unique to laboratory strains, and 43 shared between environments (Chandler et al., 2011). **(B)** For *N. vectensis*, on the other hand, there are a total of 29 associated OTUs with 19 specific to wild individuals, nine unique to laboratory strains, and one shared between environments (Har et al., 2015). These data imply that laboratory-based microbiome studies do not represent the symbiotic microbial community of the host and have large quantitative differences in associated OTUs, and thus potentially its ability to withstand, for example, environmental stressors.

Marked declines in diversity and shifts in composition, and likely function, of the microbiome follow the onset of captivity, domestication, or other anthropogenically modified environments. Decreased microbial diversity and shifts in the species present have been reported for a number of the taxa, including but not limited to insects (Figure 1A), cnidarians (Figure 1B), lizards, fish, woodrats, and other mammals (Ley et al., 2008; Fraune et al., 2010; Wang et al., 2014; Kohl et al., 2014b, 2017; Mortzfeld et al., 2015; Clayton et al., 2016; Table 1). Therefore, defining the full diversity and functional traits of the microbiome with respect to the host would benefit from considering individuals in their native ecological niches to quantify if and how these differences shape the hologenome.

Recent studies using traditional model animals (e.g., *Drosophila*: Chandler et al., 2011; *Caenorhabditis elegans*: Berg et al., 2016; Dirksen et al., 2016; Samuel et al., 2016) have addressed the limitations of the laboratory and have begun performing complementary studies with wild populations. *Drosophila*, for example, has shown to be a tractable system for characterizing the role of the host genome in mutualistic partnerships with microbes. Using 14 *Drosophila* species spanning a broad geographical distribution and in direct comparison with common laboratory strains, Chandler et al. (2011) showed that the assembly of the microbiome is primarily bound by host diet and physiology, and the degree (i.e., diversity) of this assembly is a function of the environment (laboratory vs. wild) in which the individual resides. As such, laboratory-reared Drosophilids associate with a microbiome much lower in richness and diversity than wild conspecifics. Of the 139 Operational Taxonomic Units (OTUs) found to associate with *Drosophila* spp. in these settings, 90 (64.7%) were specific to wild individuals, six (4.4%) were unique to laboratory strains, and 43 (30.9%) were shared between environments (Chandler et al., 2011; Figure 1A).

Aquatic organisms, both marine and freshwater, inhabit fluids that are particularly rich in environmental microbiota. Similar to traditional terrestrial models (e.g., *Drosophila* and *Caenorhabditis elegans*), aquatic animals are amendable to both field and laboratory experiments. A rising aquatic model species in the fields of evolutionary ecology and genomics is the estuarine cnidarian *Nematostella vectensis* (Darling et al., 2005; Tarrant et al., 2015). Recent studies using laboratory populations have shown that the microbial diversity associated with *N. vectensis* individuals is influenced significantly by developmental stage, temperature, and salinity, as well as the geographic location from which individuals were collected, despite ten years of laboratory culture (Mortzfeld et al., 2015). Like with the Drosophilids, *N. vectensis* collected from their natural habitat and subsequently cultured in the laboratory lose a significant portion of their microbial diversity and, in turn, associate with species of microbes not identified in the field (Figure 1B) (see, Figure 3 in Har et al., 2015). Comparing field and laboratory cultured individuals from the same geographical location showed that of the total 29 OTUs found to associate with *N. vectensis*, 19 (66.5%) were specific to wild individuals, nine (31.0%) were unique to laboratory strains, and one (3.4%) was shared between environments (Har et al., 2015).

Like the examples above, *Caenorhabditis elegans* taken directly from native habitats associates with a rich community of microbial symbionts (Dirksen et al., 2016), which has significant impacts on individual physiology and life-history (Cabreiro and Gems, 2013). Similarly, gut microbiota of wild mice (*Mus musculus*) are rich in diversity (Weldon et al., 2015) and dominated by *Bacteroides* and *Robinsoniella* enterotypes, a term coined by Arumugam et al. (2011) in reference to clusters of microbiota communities. Following the onset of captivity, mice were found to only maintain an association with the *Robinsoniella*-dominated enterotype (Wang et al., 2014). In another rodent species, Kohl and Dearing (2014) showed that individual desert woodrats (*Neotoma lepida*) transferred to the laboratory share 64% of their gut microbiota with individuals found in the wild. Therefore, although model organisms have historically served as exquisite laboratory-based systems for studying animal genomics and development, this approach remains more limited to study the role of the microbiome on host ecology and evolution.

In nature, holobionts face a diversity of biotic and abiotic stressors that may challenge the host to associate with a microbial community that performs an appropriate physiological response. What this implies is that when facing an ecological “task” a holobiont maximizes fitness through changes in the associated microbiota, as derived from a larger network of microbial partners, to form a complementary metabolic and physiologic profile. As stated in the hologenome theory (see, Zilber-Rosenberg and Rosenberg, 2008; Rosenberg and Zilber-Rosenberg, 2013, 2016; Bordenstein and Theis, 2015), and as presented as a conceptual extension here, a network of microbial partners would enable the holobiont, with its respective hologenome, to acclimate to short-term changes in the local environment as well as serve as a fitness landscape for adaptation (e.g., Soen, 2014). We next compare the composition of host-associated microbiota across the host's natural environments and describe implications of an associated microbial repertoire.

## Host-associated microbial repertoire

The environment is a selective filter where variation in microbial communities is sorted, resulting in the opportunity for evolutionary innovation, whether the origin of eukaryotic cells (Margulis, 1970) or the gut microbiota of invertebrates and vertebrates (Alberdi et al., 2016; Shapira, 2016). The microbial community in association with a eukaryotic host is generally considered to be a mixture of resident and transient species that vary over space and time. Regardless of the host-microbe interaction, the associated microbial community represents a small subset of the total species present in the surrounding environment (Ley R. E. et al., 2006; Lynch and Neufeld, 2015). The genomes of these microbes, which are found in the environment, a fraction of which associate with a particular host, constitute the environmental metagenome (Lynch and Neufeld, 2015). The combination of obligate and facultative microbial symbionts represents a complex set of potential interactions between host and each individual microbe as well as amongst members of the microbial community. This set of interactions is vast and discerning the type of association (i.e., mutualistic, pathogenic, neutral; Mushegian and Ebert, 2015) is an immense but important goal for determining the relative role of a microbe to the holobiont. These interactions should, in part, be dependent on the specific environment experienced by the holobiont (Steinert et al., 2000) or more indirectly due to the host's condition, which may also depend on the environment.

Recent investigations have begun resolving questions focused on how and why microbial communities shift under abiotic and/or biotic environmental stressors (e.g., Casey et al., 2015; Lokmer and Wegner, 2015; Webster and Thomas, 2016). These associations can be broadly viewed as the product of a host genome by microbial metagenome by environment interaction, or G_H_ × G_M_ × E (Bordenstein and Theis, 2015). Further determining the functional role of the microbiome in the face of environmental stressors would require manipulating E and measuring G_M_ in the context of G_*H*_. This would mean that, in some or all cases, G_H_ + G_M1_ in E_1_ differs to some degree from G_H_ + G_M2_ in E_2_. The sum of unique microbial associates (G_Mtotal_) in E_1+2_ therefore exceeds that of G_M1_ and/or G_M2_. G_Mtotal_ thus represents the microbial associates as part of the “hologenome” that exceeds either individual environment.

**Figure 2:**
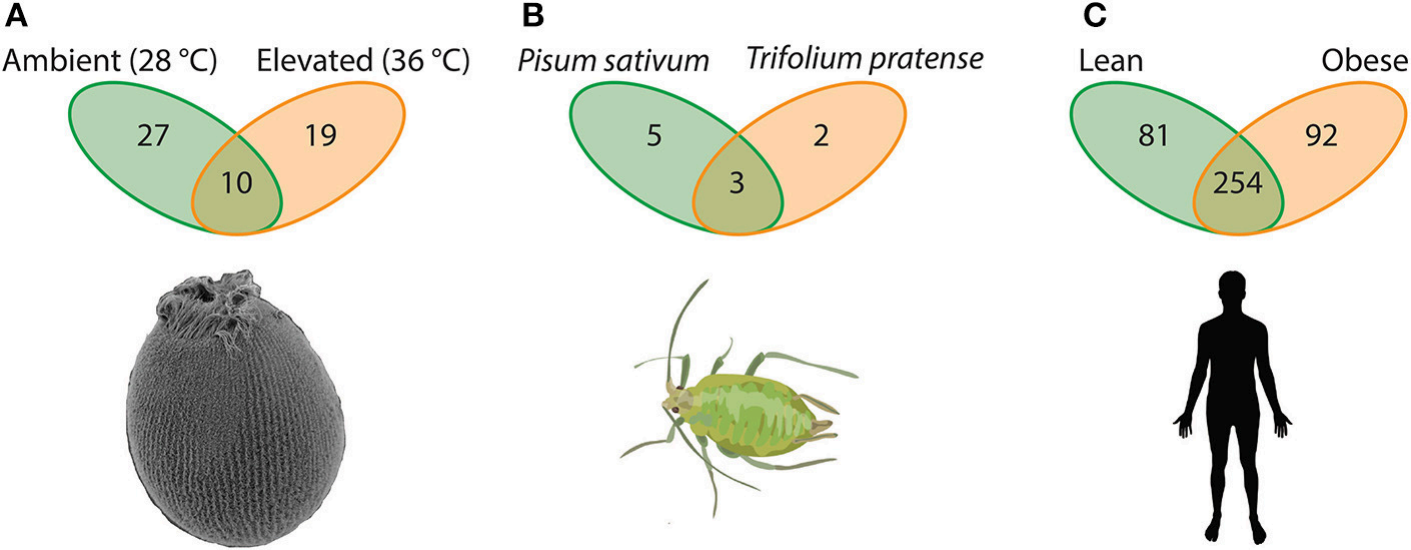
**Elements of a host-associated microbial repertoire**. Hosts in their natural setting experience a complex environment whereby abiotic and biotic factors vary spatially and temporally, and to cope with these factors the host must associated with appropriate microbial symbionts. **(A)** Larvae of the Great Barrier Reef sponge *Rhopaloeides odorabile* are exceptionally vulnerable to ocean warming. One mechanism *R. odorabile* larvae (and likely others) use to cope with temperature stress is by altering their microbial community (Webster et al., 2011a). **(B)** Aphids have adapted to effectively utilize diverse saps from plants. Some species of aphids, such as *Acyrthosiphon pisum*, exploit nutrients from multiple sap-types and, as a product, require unique microbial communities to process each sap-type (Gauthier et al., 2015). **(C)** Gut microbiota of humans provides an important metabolic complement to digest complex diet-derived biomolecules. An imbalance in the abundance and composition of these members results in a differential ability to utilize energy (Turnbaugh et al., 2006, 2008, 2009). Collectively in these three examples the total OTUs between “environments” exceeds that of an individual environment; thus, suggesting that these taxa (and likely others) have a microbial repertoire that they may associate with in a given environmental setting.

For example, when exposed to stressful temperatures, larvae of the Great Barrier Reef sponge *Rhopaloeides odorabile* lose partnerships with microbiota formed under ambient temperature (in particular the *Nitrospira, Chloroflexi* and a *Roseobacter* lineage) while forming partnerships with other microbiota (e.g., γ-proteobacteria) not previously part of the hologenome (Webster et al., 2011a). Under these two temperature regimes, *Rhopaloeides odorabile* larvae associated with 56 unique OTUs; 27 OTUs (48.2%) being specific to ambient temperature, 19 OTUs (33.9%) when faced with temperature stress, and 10 OTUs (17.9%) being shared between conditions (Webster et al., 2011a; Figure 2A). On the other hand, individual aphids (*Acyrthosiphon pisum*) specialized to the pea *Pisum sativum* as opposed to the red clover *Trifolium pratense* have five and two unique facultative associates, respectively, with three associates being shared between these diets (Figure 2B; Gauthier et al., 2015). Therefore, in these as well as other instances that span much of the animal kingdom (e.g., see Webster et al., 2011a; Gilbert et al., 2015; Har et al., 2015; Mortzfeld et al., 2015; Alberdi et al., 2016; Gilbert, 2016; Kohl and Yahn, 2016; Shapira, 2016; Webster et al., 2016; Webster and Thomas, 2016, as well as references therein; Figure 2), the collection of unique microbes as part of the hologenome in just two environmental conditions exceed that of either hologenome alone, such that by considering two environments in these focal cases (Webster et al., 2011a; Gauthier et al., 2015), the microbe associated with one host can more than double.

The properties of the environment can continually change, such that there are a nearly infinite number of unique environments, some combination of which must be taken into consideration when describing the evolutionary and ecological history of holobionts and their hologenomes. It is instructive to consider this interaction between the hologenome and the environment as G_H_ × G_M_ × E_∞_. This raises the question: what is the maximum number of microbial species a host may associate with its hologenome over the course of its life cycle in the presence and absence of all natural and anthropogenic biotic and abiotic factors? The composition of G_M_ changes with respect to E due to the acquisition and loss of “transient” microbial symbionts while G_H_ is nearly constant per generation but dynamic over evolutionary time. For simplicity, if we first consider G_H_ as a constant for a given host and that the composition of G_M_ differs with respect to E and is subsequently integrated across this continuum, then G_H_ plus the sum of G_M_, or G_H+M_, should represent the host genome plus the maximum number of microbial species a host may associate with over the course of its life-history under all encountered environmental circumstances. As such, we define this as the “host-associated microbial repertoire” (*H*):

(1) $$H=\;\int_{0}^{E}\sum^{\text{​}}{\text{G}}_{\text{H}+\text{M}\cdot }$$

Furthermore, for a given species, G_H_ is variable (e.g., Wegner et al., 2013; Mortzfeld et al., 2015; Chong and Moran, 2016) between individuals within a population and dynamic over evolutionary time, implying that the magnitude and composition of *H* may vary with respect to the interaction between G_M_ and E. Thus, each G_H_ would have a specific capacity to establish partnerships with a set number of microbial species due to the host genome and the environment (see, Sonnenburg et al., 2016).

**Figure 3:**
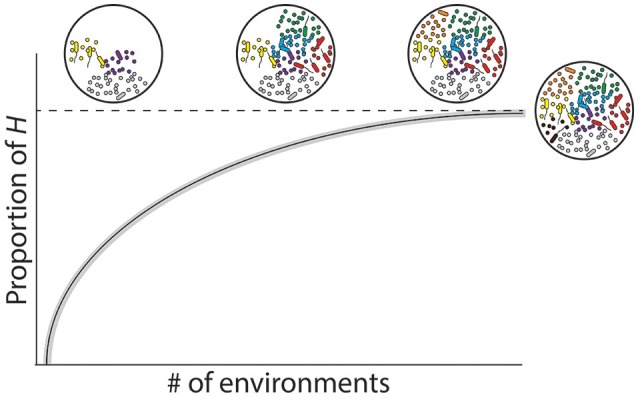
**Host-associated microbial repertoire and respective rarefaction curve**. Holobionts consist of an individual host and associated microbiota that varies due to the interactions between the host genome, microbial metagenome, and environment (G_H_ x G_M_ x E; Bordenstein and Theis, 2015). In principle, the composition of the hologenome reflects the ecological niche in which the holobiont inhabits, such that each environment may correspond with a unique hologenome. By enumerating the total unique microbial associates within an environment and then summing them across all environmental conditions in which a given host naturally encounters, we predict that the holobiont compiles its hologenome from a grander network of associated microbes, which we term the “host-associated microbial repertoire” (*H*).

The environmental factors contributing to the structure and composition of the hologenome is also influenced by time (*t*). In the context of the host-associated microbial repertoire, *t* can be represented in two primary ways: (i) absolute time of the host genome, microbial metagenome, and environment (“ecological time”) or (ii) accumulative time for co-evolution of the holobiont (“evolutionary time”). For a holobiont, absolute time is the duration of a specific cycle at each level of the G_H_ × G_M_ × E interaction, whereas the generation time for a bacterium is typically minutes to days, while that of the host could be decades. Environmental cycles, on the other hand, can encompass all of these time scales: the North Atlantic Oscillation, one of the most prominent and recurrent patterns of atmospheric variability, differs on a decadal scale (Hurrell et al., 2003), but temperature, oxygen, pH, and other abiotic factors may vary daily. Accumulative time, however, more explicitly integrates across timescales when describing chronologically distinct events over the course of development or selection of advantageous characteristics. Thus, this suggests that the repertoire of microbial species a host genome can associate with should plateau (i.e., reaching *H*) over evolutionary time based on environment-specific physiological requirements (Figure 3) (e.g., Reveillaud et al., 2014). We may now incorporate *t* into Equation 1 and, in doing so, create an equation that is conceptually analogous:

(2) $$H=\int_{0}^{t}\sum^{\text{​}}G_{M}(t)\cdot G_{H}(t)\cdot E_{t}(t)dt$$

Here, instead of integrating over E specifically, we integrate over *t*; thus, demonstrating that both *t* and E are important components driving ecological and evolutionary change of holobionts and hologenomes.

The environment experienced by the host, whether laboratory vs. field or the presence/absence of a stressor(s), may largely define the composition and structure of the associated microbiome and corresponding physiological function (Turnbaugh et al., 2009; Chandler et al., 2011; Webster et al., 2011a; Gauthier et al., 2015; Har et al., 2015). Based on the conceptual framework above, of which we view as a further development of an existing component of the hologenome theory of evolution (Zilber-Rosenberg and Rosenberg, 2008; Rosenberg et al., 2009; Bordenstein and Theis, 2015), we identify three “signatures” for applying our hypothesis: diet, indirect life cycles, and seasonality (but see Kohl and Carey, 2016 for others). In doing so, we use examples from “model” and “non-model” animals across the animal kingdom with unique life-histories that may serve to answer sets of questions pertaining to evolutionary and ecological functions of the microbiome.

### Signatures of *H*: diet

Feeding history and diet composition have a significant impact on the gut microbial communities of many animals (e.g., Ren et al., 2016) because the ability of the host to metabolize specific dietary biomolecules is dependent on the composition of these consortiums. Host species that feed on food sources that cannot be metabolized by the host or would be toxic in the absence of certain symbionts are more fit when associated with microbes that have these metabolic pathways. Thus, microbes can facilitate shifts in the permissible food sources from otherwise inaccessible energy sources (e.g., Hehemann et al., 2010), which may be a driving force in evolution of some groups (e.g., mammals; Ley et al., 2008; Alberdi et al., 2016). Microbial communities also respond to, and perhaps facilitate the host response to, prolonged periods of food deprivation (Kohl et al., 2014a) by providing the physiological toolkit that facilitates survival of the host (Turnbaugh et al., 2006, 2008, 2009; Mueller and Sachs, 2015; Kohl and Carey, 2016). In the context of the host-associated microbial repertoire, the sum of unique OTUs associated with the gut for each diet and feeding history along with the shared OTUs between them, enumerate *H*_*gut*_ for an individual at a point in time.

One of the most comprehensive systems outlining the environmental influence of feeding on the host-associated microbiome is the gut microbiota of humans. Changes in diet can result in shifts in the gut microbiome that favor a lean or obese phenotype (e.g., Ley et al., 2005; Turnbaugh et al., 2006, 2008, 2009; Ley R. et al., 2006; Spor et al., 2011). The microbiome of individuals exhibiting an obese phenotype have a higher metabolic efficiency, in part, because of a higher *Firmicutes* to *Bacteroidetes* ratio (Turnbaugh et al., 2006, 2009). These microbes plus several others make up a portion of the core gut microbiota (Turnbaugh and Gordon, 2009; Turnbaugh et al., 2009); however, the complete composition of gut flora microbes in these states extends beyond these major players. We reanalyzed the 16S metagenomic data from Turnbaugh et al. (2009) to determine how microbial OTUs are distributed between these two phenotypes. Our analysis shows that there are 427 OTUs (with four or more reads) between the gut microbiome of lean and obese phenotypes. Of these, 254 OTUs (59.5%) were shared while 81 OTUs (19.0%) were specific to the lean phenotype and 92 OTUs (21.5%) were obese-specific (Figure 2C). In a related study, Yatsunenko et al. (2012) reported that adults in the United States have upwards of 1,200 associated OTUs while Amerindian and Malwian adults have in excess of 1,400 OTUs and 1,600 OTUs, respectively, implying that Amerindian and Malwian adults have approximately 200 and 400 unique microbes in comparison to adults in the United States on a Western diet. Therefore, the gut microbiota of humans corresponds physiologically with the environmental (feeding) conditions, implying a change in associated microbiota derived from a repertoire of microbial partners. This example emphasizes the additional importance of longitudinal and regional variation in microbiomes in the evolution of hologenomes (further discussed in Zilber-Rosenberg and Rosenberg, 2008; Rosenberg and Zilber-Rosenberg, 2013, 2016).

### Signatures of *H*: indirect life cycles

Life-history strategies are diverse and many animals have successive stages occurring in unique ecological niches. Developing embryos and larvae often experience a different environment from juveniles and adults (e.g., Strathmann, 1985). Animals with biphasic life-histories provide experimental systems to discern how ecological experience influences two inter-related facets of the associated microbial community: (i) colonization of developmental stages and impacts of these on microbial communities of subsequent developmental stages, and (ii) developmental stage-specific microbial communities for different ecological niches.

The colonization of sexually reproduced offspring by microbes is dependent on mode of microbe transmission as well as the mechanisms for selection of microbial symbionts. First, the classic dichotomy of vertical and horizontal transmission of symbionts from parent to offspring results in different probabilities for successful establishment of microbes in successive generations (Bright and Bulgheresi, 2010). Whether beneficial or not, vertically transmitted microbes are more likely to contribute to the offspring's microbial community than those that are selected for from the surrounding environment. However, these transmission strategies more likely reflect a continuum dependent on maternal or paternal behaviors that may influence the likelihood of offspring being exposed to particular microbial species (Funkhouser and Bordenstein, 2013), as seen in dung beetles (Schwab et al., 2016). Second, studies using diverse animals are beginning to show how individuals initially acquire their microbiome. Two divergent strategies can occur: (i) little apparent selection for microbes followed by a winnowing in later stages (Nyholm and Mcfall-Ngai, 2004) or (ii) stricter selection for colonizers (Funkhouser and Bordenstein, 2013; Lema et al., 2014), with the latter sometimes being independent of the environmental microbial community (Apprill et al., 2012). Because the initial colonizing microbes can significantly influence the ability for later colonizing species to successfully establish on a host (Cho and Blaser, 2012), the influence of the host can be modulated by inter-microbial interactions in community establishment.

Species with biphasic life cycles, including many marine invertebrates, insects, and amphibians, would be predicted to have altered associated microbiota in response to shifts in life stage and corresponding environmental niche. Many marine invertebrates, for example, release eggs into the water column that are fertilized and develop into either planktotrophic (feeding) or lecithotrophic (non-feeding) larvae that remain in the plankton for weeks to months (Levin, 2006; Shanks, 2009) or, in some cases, more than a year (Strathmann, 1978; Strathmann and Strathmann, 2007). In terrestrial habitats, larvae are predominately in- or epifaunal and adults may be aerial and potentially more mobile (e.g., winged insects). In both environments, it is common that these life cycle stages differ in exposure to abiotic and biotic stressors. Moreover, a single life cycle stage (e.g., larvae) often experiences spatial and temporal stochasticity of abiotic factors, and specifically for feeding larvae, large shifts in the composition and availability of food (Olson and Olson, 1989).

An example of the magnitude that pre- and post-metamorphosis environments differ comes from the deep-sea mussel “*Bathymodiolus*” *childressi*. Larvae of these mussels utilize ocean currents to migrate from deep-sea methane seeps to the surface waters to feed (Sibuet and Olu, 1998; Arellano et al., 2014). Similarly, invertebrate taxa at hydrothermal vents, such as the tubeworm *Riftia pachyptila* and crab *Bythograea thermydron* disperse 10s–100s of km in search of a habitable vent site (Marsh et al., 2001; Adams et al., 2012). These examples outline the substantial difference in environments that life-history stages experience, and, in doing so, are projected to have a diverse repertoire of symbiotic microbiota corresponding to abiotic and biotic variation in each respective habitat type. If microbes were significant players in life-history evolution, then we would expect that changes in microbial species associated with animals evolving specializations in habitat, prey, etc. should have a signature when compared with other species representative of the ancestral condition.

The host-associated microbial repertoire for taxa with complex life-histories can be sub-grouped based on developmental stage, and transitioning between stages may make divisions of the host-associated microbial repertoire at one stage different than at another (*H*_*larva*_ vs. *H*_*adult*_; e.g., Wang et al., 2011). Furthermore, exposure to an environmental stress during an early life stage may later shape the initial colonizers at a later life stages and corresponding repertoire of microbial associates (e.g., amphibians: Kohl et al., 2013; insects: Hroncova et al., 2015; Chen et al., 2016; cnidarians: Fraune et al., 2016). These stage-specific influences on the physiology and survival of subsequent stages are referred to as carryover or latent effects (Pechenik et al., 1998; Pechenik, 2006). Thus, microbial communities may similarly constitute a carryover or latent effect when determining how the microbial community changes over life histories in different ecological niches and why conspecifics may vary despite experiencing similar environments.

### Signatures of *H*: seasonal variation

The diversity of ecological scenarios a host faces in its natural setting may also be driven by changes in the seasonal environment. Animals have diverse physiological and behavioral responses to the time of year that corresponds with seasonal changes, including periods elevating (e.g., reproduction and migration) and suppressing (e.g., hibernation and diapause) activity during life-history stages (Kohl and Carey, 2016). The associated microbes with a host that experience seasonal events change either in response to a new environment or in preparation for physiological shifts (Davenport et al., 2014; Kohl and Carey, 2016). One group that experiences an array of seasonally-driven ecological stressors that are also a well-studied system for animal-bacteria interactions in the marine environment (e.g., Lokmer and Wegner, 2015) are the bivalve mollusks.

Bivalves, such as mussels, clams, and oysters, inhabit the benthos throughout the world oceans, where the primary mode of energy acquisition is actively feeding on phytoplankton via pumping water from its surrounding and concentrating suspended particles (Jørgensen, 1996). The phytoplankton community changes throughout the year, particularly following the onset of the spring phytoplankton bloom, where the concentration of these particles increases by an order of magnitude of more (Evans and Parslow, 1985; Townsend et al., 1994). On a seasonal time scale, the phytoplankton community shifts from being dominated by large diatoms to small, mobile flagellates, with the zooplankton bloom lagging that of the phytoplankton bloom. As a consequence of dynamic shifts in the phytoplankton community during a bloom, it is predicted that the gut microbiota of marine bivalves will change and be constituted by more microbes with complementary metabolic and physiologic profiles for the shifted feeding regime (King et al., 2012). In the context of the host-associated microbial repertoire, we would predict that the bivalve microbial community would respond to shifts in phytoplankton composition as well as abundance, as specifically sub-divided by season (e.g., diatoms vs. dinoflagellates) and food source (e.g., phytoplankton vs. zooplankton).

The phytoplankton community does not, however, consist solely of phytoplankton beneficial for host growth and reproduction. Harmful phytoplankton produce toxins and secondary metabolites that are detrimental to health of bivalves and other animals (Hallegraeff, 1993; Anderson, 1994; Landsberg, 2002), and thus are harmful to the host and potentially the associated microbiota (Kohl and Dearing, 2012). Hosts may evolve resistance to these toxins if there is sufficient selection: in some populations of the soft-shell clam *Mya aneraria*, such as those in the Bay of Fundy, Canada, a mutation in a sodium channel that saxitoxins of the harmful dinoflagellate *Alexandrium fundyense* block arose, thereby stochastically developing a sub-population resistant to these toxins (Bricelj et al., 2005). An alternate solution to this is that bivalves acquire microbial symbionts able to metabolically utilize these potent neurotoxins. This mutation has independently arisen in at least two species of bivalves, the blue mussel *Mytilus edulis* and *Mya aneraria* (Stewart et al., 1998), through associations with *Moraxella, Alteromonas*, and *Pseudomonus*.

Association with microbes able to utilize or degrade natural toxins is not unique to marine bivalves. For example, sub-populations of woodrats (*Neotoma bryanti* and *N. lepida*) have specialized to consume the toxic creosote bush *Larrea tridentate* while other individuals in the same geographical location have not. When digesting the phenolic-rich leaves, woodrats populations that consume the toxin exhibited a marked increase in the diversity of their gut microbiota that further remained distinct from a non-toxic diet (Kohl and Dearing, 2012). Moreover, populations having had no previous exposure to phenol exhibited a decline in the diversity of gut microbiota. This, similar to the bivalve example above, suggests that microbial symbionts enable the host to adapt to consuming toxic biomaterials (Kohl and Dearing, 2012).

## Expanding theory on holobionts

**Figure 4:**
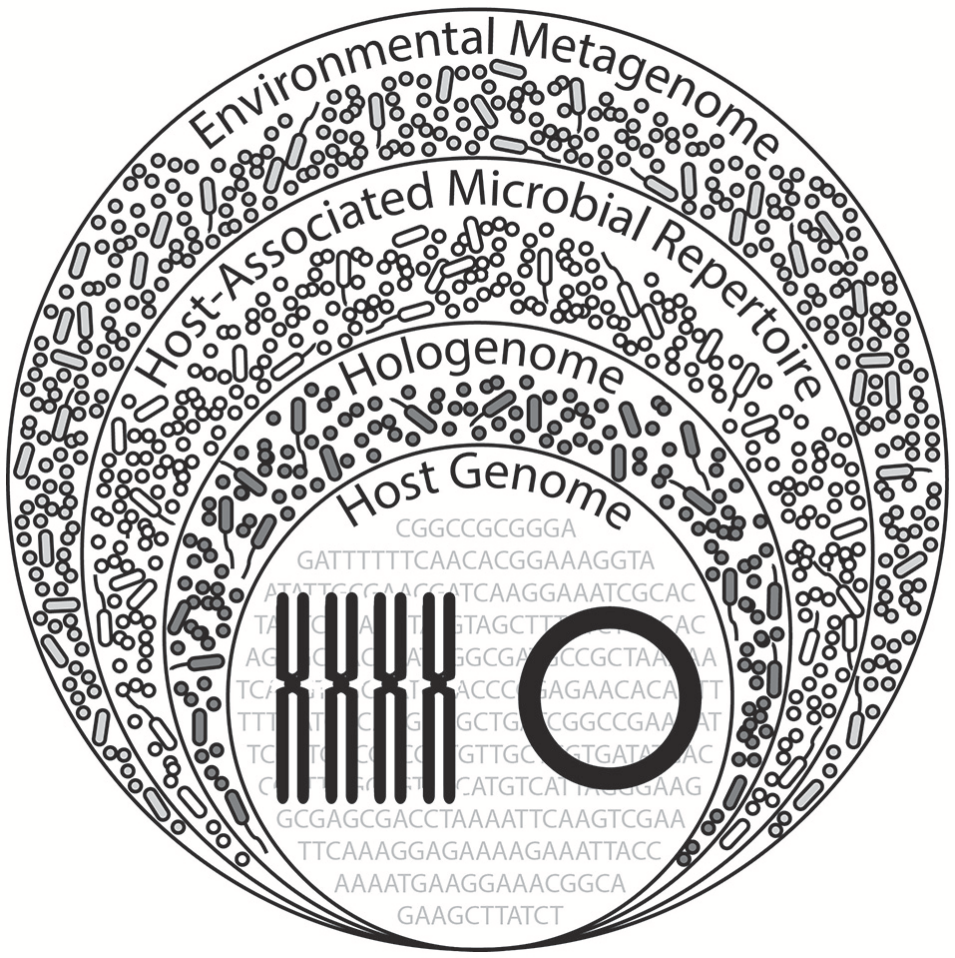
**Proposed expansion of hologenomic organization**. A hologenome comprises the total genomic components of the host (nuclear and mitochondrial genomes) and associated microbiota (bacterial, viral, archaeal, and fungal). Across diverse environments experienced by the holobiont, contents of these hologenomes exceed that of a hologenome in a single, unique environmental setting, implying an additional layer of hologenomic complexity. We term this the “host-associated microbial repertoire,” which may alternatively be viewed as the collection hologenomes associated with the host, likely holding a biological or ecological importance in the context of the environment. (This Figure was inspired by Theis et al., 2016 and subsequently expanded here).

Nearly a decade ago, Zilber-Rosenberg and Rosenberg (2008) proposed the hologenome theory of evolution, which has been summarized, expanded (Figure 4), and clarified in recent years, providing novel hypotheses for the evolution of holobionts (Bosch and McFall-Ngai, 2011; Gilbert et al., 2012, 2015; Theis et al., 2016). Current theory and research on holobionts are now beginning to explicitly consider the variation in a natural environmental context, in part, because it is a necessary factor to understand the evolution of holobionts and hologenomes (Zilber-Rosenberg and Rosenberg, 2008; Theis et al., 2016). Studies of the host-associated microbial repertoire are useful in the effort to highlight the importance of microbes in host biology (e.g., physiology, development, immunology, behavior, population genetics; Jaenike, 2012; Eisthen and Theis, 2016; Kohl and Carey, 2016; Shropshire and Bordenstein, 2016) and ecology. Furthermore, this concept may aid in advancing our understanding of how these associations may have driven or directed the evolutionary trajectory of the holobiont, and their inheritable repertoire of symbiotic microbiota (van Opstal and Bordenstein, 2015; Gilbert, 2016).

Our Hypothesis and Theory article has primarily focused on animal-associated microbiota but numerous studies suggest that plants, fungi, and other eukaryotes would have similar associations. Plant-associated microbiota (primarily bacteria and fungi) clearly have important roles in nutrient acquisition and buffering against both abiotic and biotic stressors, such that the plants are associated with particular microbiota that aid in acclimating to the local environment (Vandenkoornhuyse et al., 2015). For example, the evergreen tree *Metrosideros polymorpha* inhabits environments that broadly range in annual precipitation as well as mean temperature. In profiling the fungal endophyte communities across such abiotic gradients, the composition of these communities is directly related to temperature and rainfall (Zimmerman and Vitousek, 2012), implying that like animal examples presented through this article, plants may also associate with a larger network of microbial partners as a product of environmental variation.

As microbial taxa have been linked to specific evolutionary processes, such as *Wolbachia* and reproductive compatibility (e.g., Bordenstein et al., 2001) or *Vibrio fischeri* and countershading (e.g., Nyholm and Mcfall-Ngai, 2004), clusters of microbial associates as part of the host-associated microbial repertoire may be linked to ecological functions (Krause et al., 2006; Langille et al., 2013) including tolerance to salinity (e.g., Schmidt et al., 2015) and/or temperature (e.g., Webster et al., 2011a; Kohl and Yahn, 2016). Characterizing the host-associated microbial repertoire and subsequent function for clusters of associated microbiota may, therefore, serve as an conceptual framework that links the ecological role of microbes to the evolution of the host with which they associate. Furthermore, although these microbial associates have an ecologically important function for the holobiont, the environment in which they have the potential to associate with a holobiont may, in turn, be physiologically stressful to the microbes; thus, it is equally important to study the physiological parameters of the members of the host-associated microbial repertoire.

## Author contributions

All authors listed, have made substantial, direct and intellectual contribution to the work, and approved it for publication.

### Conflict of interest statement

The authors declare that the research was conducted in the absence of any commercial or financial relationships that could be construed as a potential conflict of interest.
